# Multi-Task Wearable Parkinson’s Disease Detection with a Pretrained Spatio-Temporal Graph Encoder and Task-Level Token Aggregation

**DOI:** 10.3390/bioengineering13080860

**Published:** 2026-07-25

**Authors:** H. M. K. K. M. B. Herath, Nuwan Madusanka, Chaminda Hewage, Byeong-Il Lee

**Affiliations:** 1 Industry 4.0 Convergence Bionics Engineering, Pukyong National University, Busan 48513, Republic of Korea; kasunkh@pukyong.ac.kr; 2 Digital Healthcare Research Center, Pukyong National University, Busan 48513, Republic of Korea; nuwanv@pknu.ac.kr; 3 Cardiff School of Technologies, Cardiff Metropolitan University, Cardiff CF23 6PS, UK; chewage@cardiffmet.ac.uk; 4 Division of Smart Healthcare, College of Information Technology and Convergence, Pukyong National University, Busan 48513, Republic of Korea

**Keywords:** Parkinson’s disease, wearable inertial sensors, spatio-temporal graph convolutional networks, transfer learning, pretrained motion embeddings, multi-task gait analysis, transformer aggregation

## Abstract

Wearable inertial measurement units (IMUs) offer an objective, low-cost basis for Parkinson’s disease (PD) assessment, but multi-task clinical protocols yield heterogeneous recordings across body locations and small cohorts, and it is unclear whether such data can support reliable PD detection without training deep models from scratch. We therefore ask whether a motion-pretrained representation transfers to this setting, and quantify how much of the discriminative signal it supplies. Each subject is represented by five task-level motion embeddings, one per clinical task, produced by a frozen pretrained spatio-temporal graph convolutional network (ST-GCN) that fuses the thirteen body-worn sensors into a whole-body embedding; a three-layer Transformer with validity-mask weighting aggregates these tokens for binary PD-versus-control classification on the WearGait-PD cohort (181 subjects: 100 PD, 81 controls). Under a leakage-free nested protocol with repeated subject-disjoint stratified 5-fold cross-validation (5 seeds; 25 estimates per model) and paired significance testing, the model attains a balanced accuracy of 0.834 ± 0.087, macro-F1 of 0.842 ± 0.094, and AUC of 0.842 ± 0.103. It leads six classical baselines and a spectrogram-CNN on accuracy-based metrics, though random forest, gradient boosting, and the spectrogram-CNN edge ahead on AUC; after correction for fold correlation, none of these between-model differences is significant. The one robust finding is a transfer effect: replacing the pretrained encoder with a random one of identical architecture lowers balanced accuracy by 15.5 points when frozen (*p* = 0.043) and 20.4 when trained end-to-end (*p* = 0.014). Discrimination is preserved under 1:1 age matching (0.846) and across both genders, so it is not explained by age imbalance. Motion-pretrained skeletal encoders thus supply the majority of the discriminative signal, while the aggregator contributes gains inseparable from noise at this cohort size.

## 1. Introduction

Parkinson’s disease (PD) is a progressive neurodegenerative disorder characterized by bradykinesia, rigidity, resting tremor, and postural instability [1,2]. Although clinical diagnosis is anchored to the structured neurological examination summarized by the Movement Disorder Society–Unified Parkinson’s Disease Rating Scale (MDS-UPDRS) [3], the resulting scores are coarse, examiner-dependent, and difficult to administer at the frequencies required for longitudinal monitoring. Wearable inertial measurement units (IMUs) have therefore attracted sustained interest as an objective, low-cost substrate for continuous assessment of motor symptoms [4,5,6,7], capturing tri-axial acceleration and angular-velocity signals that carry measurable signatures of PD bradykinesia, tremor, and gait asymmetry [8,9,10].

Two complications make IMU-based PD detection less straightforward than this premise suggests. First, modern protocols deliberately combine several short clinical tasks, steady-state gait, transitions and dual-tasking, dynamic stability, and postural control, because each probes a distinct facet of motor control [11,12,13,14]; models that pool windows across tasks implicitly assume task-invariant PD signatures, which do not hold. Second, sensor placement is heterogeneous: in the WearGait-PD dataset used here [15], 13 body-worn sensors span the trunk and upper and lower limbs, which motivates representations that preserve the clinical-task structure rather than collapsing it. Compounding both issues, PD cohorts are typically small, on the order of one hundred subjects per group [15,16,17], which makes training Transformer-class models from scratch fragile and favors reusing foundation representations of human motion. Spatio-temporal graph convolutional networks (ST-GCNs) [18,19,20] offer a natural inductive bias for body-worn IMU data, treating the body as a graph of articulated joints whose trajectories are smoothed by convolution over the skeletal adjacency, and encoders pretrained on large motion corpora yield transferable representations adaptable to downstream wearable tasks under limited supervision [21,22,23].

We therefore build on a frozen pretrained ST-GCN encoder and pose a specific question rather than proposing a new architecture as an end in itself: how much of the discriminative signal for multi-task wearable PD detection is supplied by the transferred motion representation, as opposed to the trainable components built upon it. Because the encoder operates on the whole skeleton in each forward pass, fusing the thirteen sensors into a single whole-body embedding per task, each subject is represented by five task-level embeddings. A three-layer Transformer aggregator [24] with a learnable CLS token and validity-mask weighting maps these tokens to a subject-level representation for binary classification; the full methodology is given in Section 3. The model is evaluated on the WearGait-PD cohort [15] under subject-disjoint stratified 5-fold cross-validation, with three controlled ablations isolating the pretrained weights, the positional embeddings, and the cross-token attention, and is compared against seven baselines, a spectrogram-CNN adapted from Sassi et al. [25] and six classical learners [26,27,28,29,30] trained on the UniMTS ST-GCN embeddings, on identical splits and features.

The objectives of this study are threefold: (i) to quantify, by controlled ablation, how much of the discriminative capacity for multi-task wearable PD detection is supplied by the transferred motion representation relative to the trainable components built upon it; (ii) to benchmark the resulting model against strong classical and deep baselines on identical subject-disjoint folds under repeated cross-validation with paired significance testing; and (iii) to establish whether any observed discrimination persists once the cohort’s age and gender imbalance is accounted for. We hypothesize that the pretrained motion representation, rather than the task-level aggregator, is the primary source of discriminative signal at this cohort size.

## 2. Related Work

### 2.1. Wearable Sensing for Parkinson’s Disease

Wearable IMU sensing for PD has progressed from hand-crafted feature pipelines feeding classical learners to end-to-end deep models on raw windows. Early work focused on cadence, stride length, stride variability, and tremor-band power computed from a few sensors, typically lumbar or wrist [4,5,6,7,9,10,31], and these features remain among the most validated quantitative PD biomarkers [11,12,32,33]. End-to-end deep models have since shown that representations learned directly from raw IMU windows can match or exceed hand-crafted pipelines, particularly when window-level features are aggregated to the subject level [34,35,36,37]. The principal methodological caveat across this literature is the need for subject-disjoint cross-validation: window-level splitting reliably overestimates performance, because windows from the same subject share gait phenotype and sensor-placement noise [35].

Recent work has increasingly framed wearable IMU analysis for PD as a representation-learning problem. Beyond hand-crafted pipelines, machine- and deep-learning approaches now target transfer learning from large motion corpora, graph-based modeling of body-worn sensor arrays, foundation models for human motion, automated extraction of digital motor biomarkers, and multimodal fusion of inertial with complementary signals [38,39]. These developments motivate the transfer-learning approach adopted here: rather than proposing a further bespoke architecture, we quantify how much discriminative capacity a pretrained spatio-temporal graph representation supplies for multi-task PD detection, and how the present framework differs from and extends these recent directions.

Table 1 positions the present study among recent binary PD-versus-control detection studies using wearable inertial or pressure sensors. Cohorts, sensor configurations, and validation protocols differ across studies, so the values are not directly comparable; the table is intended to situate the contribution rather than to claim a like-for-like ranking. To our knowledge, this is the first study to report binary PD detection on the full WearGait-PD cohort using the body-worn IMU array; the only prior WearGait-PD classification work [40] addresses a single sub-task (TUG) and a single modality (pressure insoles) on a 77-subject subset.

### 2.2. Graph Neural Networks for Skeletal and Sensor-Based Motion

Yan et al. [18] established the body-graph view of human motion as a strong inductive bias for action recognition, and adaptive and two-stream variants such as 2s-AGCN [19] and CTR-GCN [36], together with shift-based [37] and channel-wise topology refinements, have since improved accuracy and parameter efficiency on action benchmarks. This view is attractive for wearable IMU data, since sensors are placed at anatomical landmarks that map naturally to skeleton joints, allowing a pretrained encoder to ingest IMU streams with minimal architectural change [22,23]. Foundation models trained on heterogeneous motion-capture corpora provide a useful starting point for downstream wearable applications when the cohort is small [21].

### 2.3. Transformer Attention and Classical Baselines in Clinical ML

The Transformer [24] is now the standard tool for set-structured reasoning, with pre-norm variants preferred for stable training [44]. In small-cohort clinical settings, deep architectures do not automatically outperform tuned gradient-boosting or random-forest baselines on tabular features [28,30,45], and a growing methodological consensus holds that strong classical baselines should be reported alongside deep models, so that a Transformer’s added capacity is shown to be accuracy-justified rather than assumed [45]. We follow this protocol, treating classical machine learning as an honest benchmark and reporting deep and classical results on identical subject-disjoint folds and features.

### 2.4. Research Gap and Motivation

Two gaps in the literature reviewed above motivate the present study. First, prior wearable-PD deep models either pool windows across tasks [34,35,36,37] or, in the only previous WearGait-PD classification study [40], restrict attention to a single sub-task and a single sensor modality; the multi-task structure of modern clinical protocols is therefore rarely preserved in the learned representation. Second, although motion-pretrained encoders are increasingly available, the extent to which such transferred representations, rather than the trainable components built upon them, account for discriminative capacity in small wearable-PD cohorts has not been quantified.

The present work addresses these gaps by keeping the clinical-task axis explicit, representing each subject as five task-level motion embeddings produced by a frozen motion-pretrained ST-GCN encoder, and measuring the transfer effect by controlled ablation under a leakage-free evaluation protocol. Because the pretrained encoder fuses the thirteen body-worn sensors into a single whole-body embedding at its joint-pooling stage (Section 3.3), the task-level embedding, rather than a task-by-sensor unit, is the appropriate unit of representation; genuinely sensor-specific modeling would require per-sensor encoding and is identified as future work (Section 5.4). A detailed discussion of how these design choices advance beyond prior work is reserved for the Discussion and Conclusions.

## 3. Materials and Methods

### 3.1. Dataset

**Figure 1:**
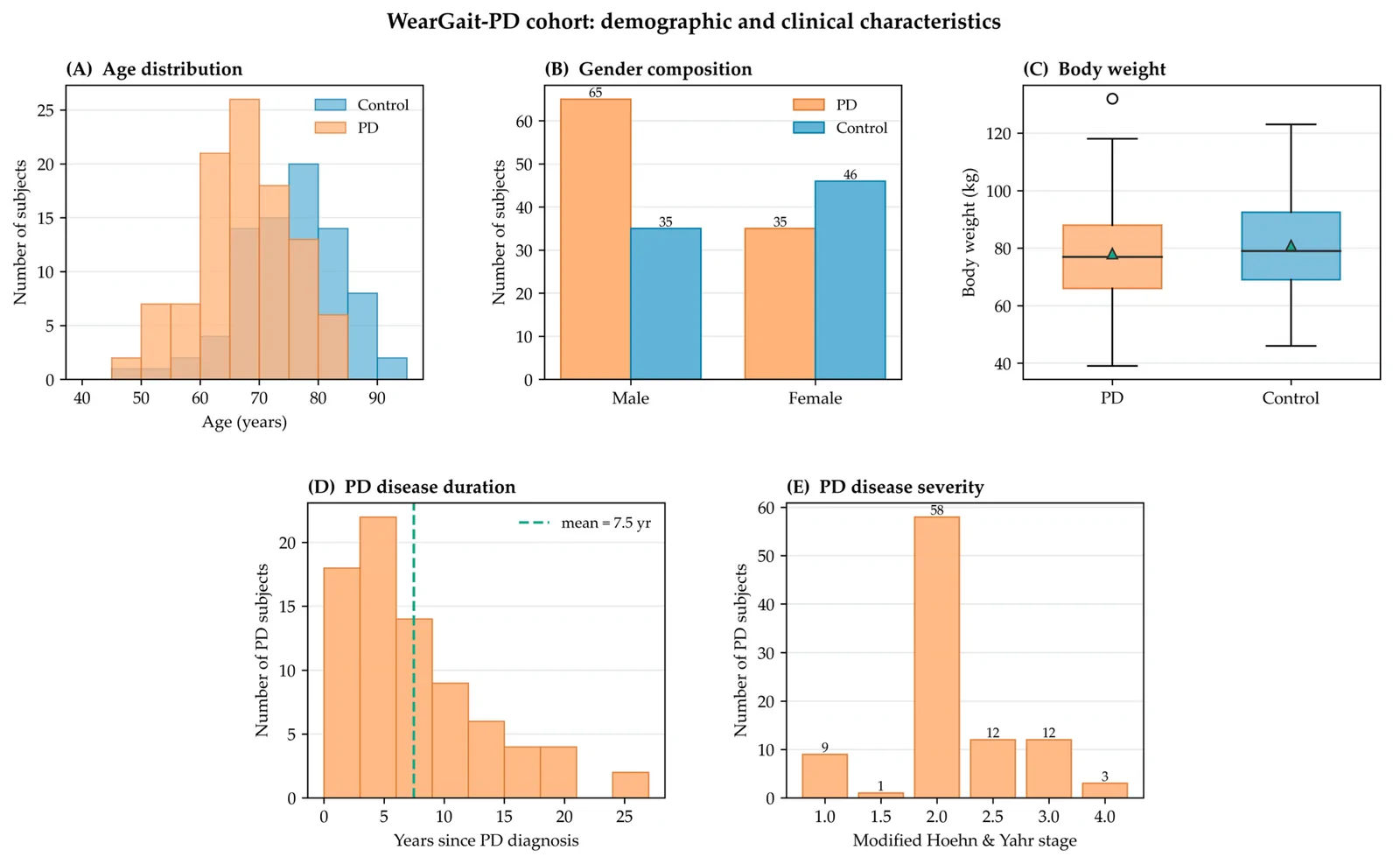
Demographic and clinical characteristics of the WearGait-PD cohort (100 PD, 81 controls). (**A**) Age distribution. (**B**) Gender composition. (**C**) Body weight, with triangles marking means; the open circle is a single PD outlier (>1.5 × IQR above the box, ≈130 kg). (**D**) PD disease duration (dashed line: mean). (**E**) PD disease severity (modified Hoehn and Yahr stage). Numerical summary in Table 2.

We use the publicly released WearGait-PD dataset [15], comprising older adults with and without Parkinson’s disease instrumented with thirteen Xsens DOT IMUs (Movella Inc., Henderson, NV, USA) sampled at 100 Hz by the original data providers. After cache reconstruction and subject-level filtering, our experiments use 181 subjects (100 PD, 81 controls). The cohort’s demographic and clinical characteristics are summarized in Figure 1 and Table 2: the two groups are broadly comparable in body weight, differ moderately in gender composition (PD 65/35 vs. control 35/46 M/F), and differ in age: PD subjects are on average younger than controls (67.0 ± 8.3 years vs. 74.7 ± 8.6 years) and span a wide range of disease durations (7.5 ± 5.9 years) and severities (modified Hoehn and Yahr stages 1.0–4.0, with the majority at stage 2.0). Although the dataset providers describe the controls as age-matched at the full-release level, in the 181-subject subset analyzed here, the control group is, on average, approximately seven years older than the PD group; age is therefore a potential confound. We address it directly rather than only in the Limitations: Section 4.4 reports a 1:1 age-matched analysis, age-stratified subgroups, and within-class probability-age correlations, together with a per-gender evaluation for the gender imbalance, and finds that discrimination is preserved under matching and across both genders.

**Figure 2:**
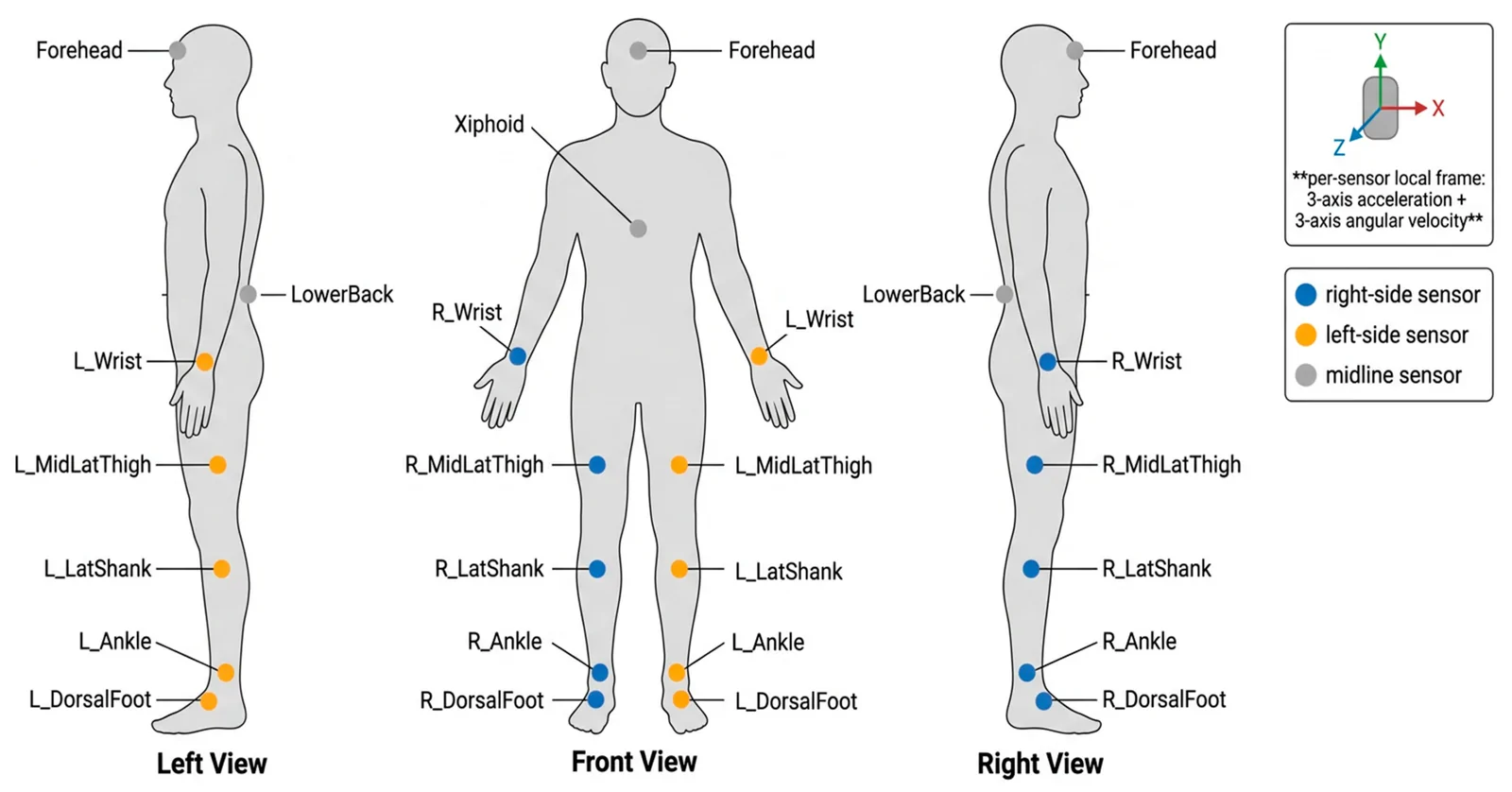
Anatomical placement of the 13 body-worn inertial measurement units in left lateral, frontal, and right lateral views. Three sensors lie on the body midline (Forehead, Xiphoid, and LowerBack); the remaining ten form five bilateral pairs at the wrist, mid-lateral thigh, lateral shank, ankle, and dorsal foot. Blue and orange markers denote right- and left-side sensors, respectively (relative to the subject’s body); the posterior LowerBack sensor appears only in the lateral views. The inset defines the per-sensor local coordinate frame; each unit records 3-axis free acceleration, raw acceleration, and angular velocity.

Each subject performs up to five short clinical tasks: self-paced walking, the TUG test, hurried-pace walking, tandem gait, and quiet-standing balance. The 13 inertial measurement units are distributed over the head, trunk and both limbs; their anatomical placement and the per-sensor coordinate frame are shown in Figure 2. Each sensor produces nine channels per sample (3-axis free acceleration, 3-axis raw acceleration, and 3-axis angular velocity). Task coverage in our cohort is summarized in Table 3.

The WearGait-PD dataset was selected because it is a public, openly documented dataset, collected with institutional review board approval by its original providers, that pairs a standardized thirteen-IMU body-worn array with a multi-task protocol covering 98–100% of subjects per task properties well matched to the multi-task, whole-body representation studied here. Its adequacy is nonetheless bounded: 181 subjects are modest for deep learning, and the release contains a single visit per subject from a single recording site, with a binary PD-versus-control labeling and no disease-severity or differential-diagnosis information.

### 3.2. Preprocessing and Windowing

Raw IMU signals were segmented per task into fixed-length 2 s windows (200 samples at the native 100 Hz rate), each carrying per-sensor validity flags; the 2 s duration matches the spectrogram baseline. The full nine-channel representation was retained at the cache stage. No band-pass filtering, drift correction, or magnetometer-based orientation correction was applied: the encoder consumes gravity-removed free acceleration, which addresses the dominant low-frequency component, and its rotation-invariant pretraining makes further orientation correction unnecessary. Missing tasks are handled through the binary validity mask, which zero-fills absent tokens and excludes them from both the standardizer and the downstream attention, so no imputation is performed. Feature standardization is applied per fold: a per-feature standardizer fitted only on non-zero training tokens is applied to held-out subjects, with the mask reapplied so absent tokens remain exactly zero (Section 3.4, Equations (2) and (3)). Resampling from 100 Hz to the encoder’s native 20 Hz (Fourier-based, with wrap-padding to 200 frames) was performed only when feeding the frozen backbone, leaving the cached full-rate signals available to the baselines. Subject identifiers were preserved end-to-end so that 5-fold cross-validation operated at the subject level rather than the window level [35], eliminating leakage from the same-subject windows appearing in both the training and test sets.

### 3.3. Token Construction

The proposed model represents each subject by five task-level tokens, one per clinical task, each a 512-dimensional motion embedding from the frozen encoder, along with a length-5 binary validity mask that flags the tasks performed. The representation has no sensor axis: the encoder fuses the thirteen body-worn sensors into a single whole-body embedding at its joint-pooling stage, so a task-by-sensor representation could only be formed by replicating the task embedding across sensors, adding no sensor-specific signal. The model, therefore, aggregates the five task tokens directly.

**Figure 3:**
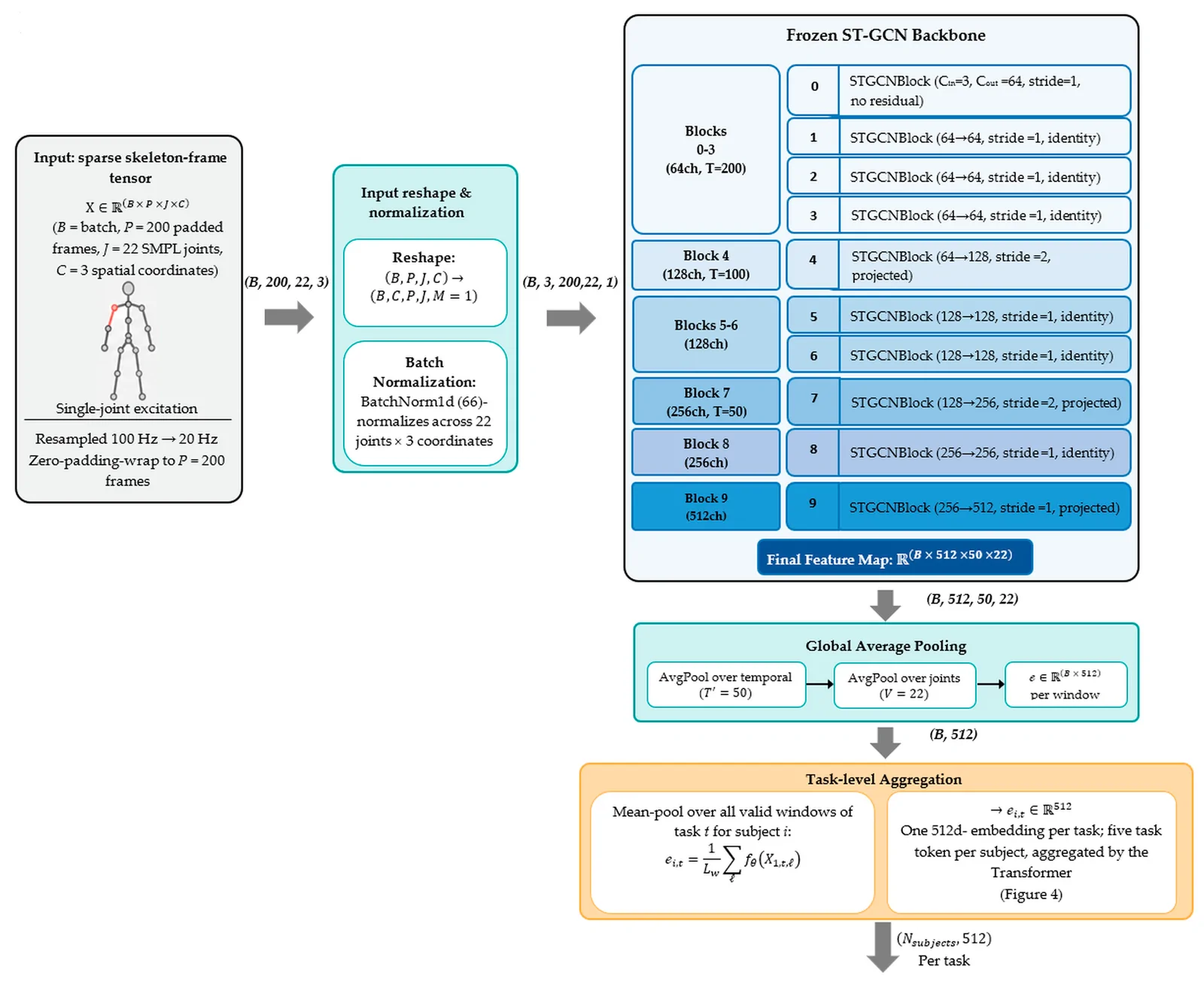
Internal architecture of the frozen, pretrained ST-GCN backbone that produces the task-level motion embedding. A sparse skeleton-frame tensor is processed by spatio-temporal graph convolutions over the 22-joint skeletal adjacency, with global pooling yielding a 512 d per-window embedding. These are mean-pooled across the valid windows for each task to yield the five 512 d task tokens consumed by the aggregator in Figure 4. The thirteen sensors are fused here, at the encoder’s joint-pooling step; no sensor axis exists downstream of it.

Task-level motion embedding (512 d). For each task, all valid windows are passed through a frozen pretrained skeletal-motion graph encoder following the ST-GCN architecture [18,21]. The pretrained backbone is the publicly released UniMTS encoder [21], obtained from the authors’ repository (https://github.com/xiyuanzh/UniMTS, accessed on 20 February 2026); the released pretrained checkpoint was used without modification. The encoder operates on a fixed 22-joint skeletal graph; this topology is intrinsic to the pretrained backbone, since the graph adjacency and learned edge-importance weights are defined over exactly 22 joints, and is not a free design choice. Because WearGait-PD provides 13 IMUs rather than a full optical skeleton, we adopt the sparse-skeleton input scheme for which this encoder class is designed: each sensor is mapped to its anatomically closest joint (Table 4), the corresponding joint channels are populated, and the remaining 9 joints are zero-filled in every window. This is the encoder’s intended usage rather than a limitation of our pipeline: the same backbone is pretrained and evaluated on configurations that populate as few as one joint; the spatial graph convolution propagates information from populated joints across the skeleton via the adjacency; and the edge-importance weighting attenuates inactive edges. Windows are resampled from 100 Hz to 20 Hz and wrap-padded to 200 samples. The encoder yields one 512-dimensional embedding per window, then mean-pools across the task’s valid windows to produce one task-level embedding per subject. The encoder’s internal structure is detailed in Figure 3.

Channel-to-coordinate mapping and its caveat. The encoder ingests a three-coordinate tensor per joint, X ∈ ℝ (P × J × 3), the same three-channel-per-joint input format that the encoder consumes during pretraining. The UniMTS encoder [21] is pretrained not on raw 3D joint positions but on physics-simulated inertial signals derived from motion-capture skeletons, using rotation-invariant augmentation that makes it agnostic to device-mounting orientation; the measured IMU streams are therefore well matched to its pretraining input. Each unit reports tri-axial free acceleration, tri-axial raw acceleration, and tri-axial angular velocity in its own local sensor frame, and in this work, the three per-joint input channels are populated with the 3-axis free-acceleration channels of the mapped sensor, since free acceleration (gravity removed) most directly reflects the joint’s motion; the remaining six channels per sensor (tri-axial raw acceleration and tri-axial angular velocity) are not consumed by the present model, which uses only the three free-acceleration channels per sensor as the encoder input.

This use of the encoder is a substitution rather than a calibrated kinematic model: it serves as a fixed, transferable feature extractor, and two mismatches relative to its pretraining persist, the local sensor frame of each IMU, only partly absorbed by the encoder’s rotation-invariant pretraining, and the use of only the three free-acceleration channels rather than the full inertial set. The substantial drop in performance when the pretrained weights are removed indicates that the resulting embedding is nonetheless discriminative. The magnitude of this residual frame-and-channel mismatch, and whether populating all available inertial channels (raw acceleration and angular velocity) would improve transfer, cannot be resolved from the present experiments and is identified as future work.

Mean-pooling the encoder output at the task level, rather than per sensor, follows from the encoder’s design: it operates on the full skeleton in each forward pass and pools globally over time and joints, so its output is already a whole-body representation conditioned on the task, with the thirteen sensors fused inside the encoder rather than downstream of it. Consequently, a representation indexed jointly by task and sensor could only be obtained by replicating this whole-body embedding across the sensors present for each task; the resulting sensor axis would record which body locations contributed to a recording, through the validity mask and a sensor positional embedding, but would carry no sensor-specific signal. The model, therefore, aggregates the five task-level tokens directly, and absent tasks are represented by zero tokens with zeroed validity-mask entries so that downstream attention ignores them. A genuinely sensor-specific representation would require per-sensor encoding rather than a replicated whole-body embedding; this is identified as future work (Section 5.4) and is also the route through which a physical sensor-reduction study could be pursued.

### 3.4. Mathematical Formulation

The proposed model maps each subject’s set of five task-level tokens to a binary prediction through the components defined below; we also specify the three component ablations and the classical-baseline pipeline evaluated against it. Throughout, *i* indexes subjects, $t\in \left\{1,\ldots ,\;T\right\}$ clinical tasks, and $l\in \left\{1,\ldots ,\;L_{w}(i,t)\right\}$ the valid windows of task *t* for subject *i*. The encoder input dimension is $d_{e}=512$ and the Transformer working dimension is *d* = 128.

- Task-level motion embedding: Each IMU window is remapped onto the *J*-joint skeleton by the sensor-to-joint assignment of Section 3.3, producing a sparse skeleton-frame tensor ${Χ}^{\left(i,t,l\right)}\in R^{\left(P\times J\times 3\right)}$ after resampling to 20 Hz and zero-pad-with-wrap to *P* = 200 frames. The frozen encoder $f_{\theta }$ maps each window to a 512-dimensional vector, and the task-level embedding is the mean over the $L_{w}(i,t)$ valid windows of task *t*:
  (1) $$z_{i,t}=\frac{1}{L_{w}\left(i,t\right)}\sum_{l=1}^{L_{w}\left(i,t\right)}f_{\theta }\left({Χ}^{\left(i,t,l\right)}\right),\;\;t\in \left\{1,\ldots ,\;T\right\}$$
  If subject *i* has no valid window for task *t*, $z_{i,t}$ is set to the zero vector and the corresponding validity-mask entries are zero, so downstream attention ignores them.
- Subject representation: Because the frozen encoder returns one whole-body embedding per task (Equation (1)), the complete representation of subject *i* is the ordered set of its five task-level embeddings {z_i,1_, …, z_i,T_}, for which we write u_i_(*t*) ≡ z_i,t_ in the equations of Section 3.4, together with a length-*T* binary validity mask m_i_ whose entry m_i_(*t*) is 1 when subject *i* performed task *t* and 0 otherwise. The model, therefore, aggregates the five task tokens directly; it does not form a task-by-sensor grid, since the encoder has already fused the thirteen sensors into each whole-body embedding at its joint-pooling stage (Section 3.3), so a replicated sensor axis would carry no sensor-specific signal.
- Per-fold standardization: For cross-validation fold *k*, let $I_{k}^{tr}$ be its training subjects. A per-feature standardizer is fitted only on the non-zero training tokens:
  (2) $${\hat{\mu }}_{k}=\underset{i\in \;I_{k}^{\mathrm{tr}},\left(t\right):\;m_{i}\left(t\right)=1}{\mathrm{mean}}u_{i}\left(t\right),\;\;\;{\hat{\sigma }}_{k}=\underset{i\in \;I_{k}^{\mathrm{tr}},\left(t\right):\;m_{i}\left(t\right)=1}{\mathrm{std}}u_{i}\left(t\right)\;$$
  and applied to every token of every subject in fold:
  (3) $${\tilde{u}}_{i}\left(t\right)=m_{i}\left(t\right)\cdot \left(u_{i}\left(t\right)-{\hat{\mu }}_{k}\right)⊘{\hat{\sigma }}_{k},$$
  where ⊘ is element-wise division. The multiplicative mask in Equation (3) keeps absent task tokens exactly zero after standardization, so attention is not biased by the standardizer’s offset on those positions.
- Token projection and positional encoding: Each standardized token is projected to the Transformer working dimension d=128 by a LayerNorm–Linear–GELU–Dropout block, with learned task-indexed positional embeddings added:
  (4) $$h_{i}\left(t\right)=Dropout\left(GELU\left(W_{p}LN\left({\tilde{u}}_{i}\left(t\right)\right)+b_{p}\right)\right)+e_{t}^{task}$$
  where $W_{p}\in R^{{d\times d}_{u}}$, $b_{p}\in R^{d}$, and $d_{e}=512$ is the encoder output dimensions, and the learned task-embedding matrix $E^{task}\in R^{T\times d}$ supplies the row $e_{t}^{task}$.
- Validity-mask token weighting: Tokens corresponding to tasks the subject did not perform are down-weighted to zero by a normalized validity weight before they enter the Transformer:
  (5) $$w_{i}\left(t\right)=\frac{m_{i}\left(t\right)}{\sum_{t^{\prime }}m_{i}\left(t^{\prime }\right)},\;\;\;\;\;\;\;\;\;\;\;\;{\tilde{h}}_{i}\left(t\right)=w_{i}\left(t\right)h_{i}\left(t\right).$$
  This weighting carries no learnable parameter and makes the marginal contribution of the present tokens independent of how many tasks are missing for that subject.
- Cross-token Transformer encoder: Let ${\tilde{H}}_{i}\in R^{T\times d}$ be the row-stacked matrix of weighted projected tokens, and let $h^{CLS}\in R^{d}$ be a learnable subject-shared CLS token. The encoder input is
  (6) $$H_{i}^{\left(0\right)}=\left[h^{CLS};{\tilde{H}}_{i}\;\right]\in R^{\left(T+1\right)\times d},$$
  with a binary key-padding mask $k_{i}\in {\left\{0,1\right\}}^{T+1}$ defined by $k_{i}\left(0\right)=0$ and $k_{i}\left(\tau \right)=1-m_{i}\left(\tau \right)$ for τ≥1, so absent tokens cannot be attended to. The encoder consists of L=3 pre-norm Transformer blocks with H=4 heads each. Block l updates the representation as
  (7) $${\hat{H}}_{i}^{\left(l\right)}=H_{i}^{\left(l-1\right)}+MHSA\left({LN}_{1}\left(H_{i}^{\left(l-1\right)}\right),k_{i}\right),$$
  (8) $$H_{i}^{\left(l\right)}={\hat{H}}_{i}^{\left(l\right)}+MLP\left({LN}_{2}\left({\hat{H}}_{i}^{\left(l\right)}\right)\right),$$
  where MHSA is multi-head self-attention with key-padding mask $k_{i}$, and MLP is the standard two-layer feed-forward block with GELU activation, expansion factor four, and dropout 0.3. The CLS embedding is the final row 0 of the last block:
  (9) $$c_{i}=H_{i}^{L}\left[0,:\;\right]\in R^{d},$$
- Classification head and loss: A two-layer MLP head produces logits over the two classes:
  (10) $${\hat{y}}_{i}=W_{2}GELU\left(W_{1}LN\left(c_{i}\right)+b_{1}\right)+b_{2}\in R^{2},$$
  with $W_{1}\in R^{d/2\times d}$ and $W_{2}\in R^{2\times d/2}$. The model is trained with class-weighted softmax cross-entropy:
  (11) $$L=-\frac{1}{N_{b}}\sum_{i\in batch}\alpha_{yi}\;log\left({softmax\left({\hat{y}}_{i}\right)}_{yi}\right),\;\;\;\;\;\;\;\;\;\;\;\alpha_{c}=\frac{N}{2\left|\left\{i:y_{i}=c\right\}\right|}$$
  where N is the number of training subjects, Nb the batch size, and the class weights $\alpha_{c}$ are computed once per training fold from the training labels.
- Cross-token attention extraction: The model also admits an attention-based interpretability readout: the CLS row of the post-softmax attention matrix is averaged across the H heads and the L encoder layers, and renormalized over the present tasks. For layer *l*:
  (12) $$A_{i}^{\left(l\right)}\left(t\right)=\frac{1}{H}\sum_{h=1}^{H}softmax{\left(\frac{q_{0}^{\left(l,h\right)}K^{\left(l,h\right)⊤}}{\sqrt{d_{h}}}\right)}_{\left(t\right)}\;,$$
  (13) $${\tilde{A}}_{i}\left(t\right)=\frac{m_{i}\left(t\right)\cdot \frac{1}{L}\sum_{l=1}^{L}A_{i}^{\left(l\right)}\left(t\right)}{\sum_{t^{\prime }}m_{i}\left(t^{\prime }\right)\cdot \frac{1}{L}\sum_{l=1}^{L}A_{i}^{\left(l\right)}\left(t^{\prime }\right)}\;,$$
  so that ${\tilde{A}}_{i}\in {\left[0,1\right]}^{T}$ and $\sum_{t}{\tilde{A}}_{i}\left(t\right)=1$ for every subject i. Equations (12) and (13) define the attention map as a byproduct available from the trained model; a population-level map can be obtained by averaging ${\tilde{A}}_{i}$ over the held-out subjects of each fold and then across folds. A clinical interpretation of this map, relating attention weight to specific clinical tasks and to known PD motor signatures, is deferred to future work, since a credible interpretation requires the repeated-seed runs described there to establish that the attention pattern is stable across resampling rather than an artifact of a single split.

### 3.5. Proposed Model Architecture

The proposed model passes each of the five task tokens through a LayerNorm–Linear–GELU–Dropout projection that maps 512 dimensions to 128, then adds a learned task-position embedding of size 128 (5 entries). Tokens are scaled by a normalized validity weight so that absent tasks contribute zero to subsequent attention, and present tasks are weighted uniformly. A learnable CLS token is prepended, and the resulting six tokens are passed through a three-layer Transformer encoder [24,44] with four attention heads, an inner feed-forward dimension of 512, GELU activation, pre-LayerNorm, and dropout 0.3. These settings follow standard small-Transformer practice and keep the trainable head small (~0.68 M parameters) for the 181-subject cohort, thereby limiting overfitting. Because the positional-embedding and attention ablations move balanced accuracy by amounts within the fold-to-fold noise (Section 4), no hyperparameter search was performed; at this cohort size, such a search would risk overfitting the evaluation, so the head is presented as parameter-efficient rather than tuned. A padding mask derived from the validity mask is supplied, preventing zero tokens from contaminating attention.

**Figure 4:**
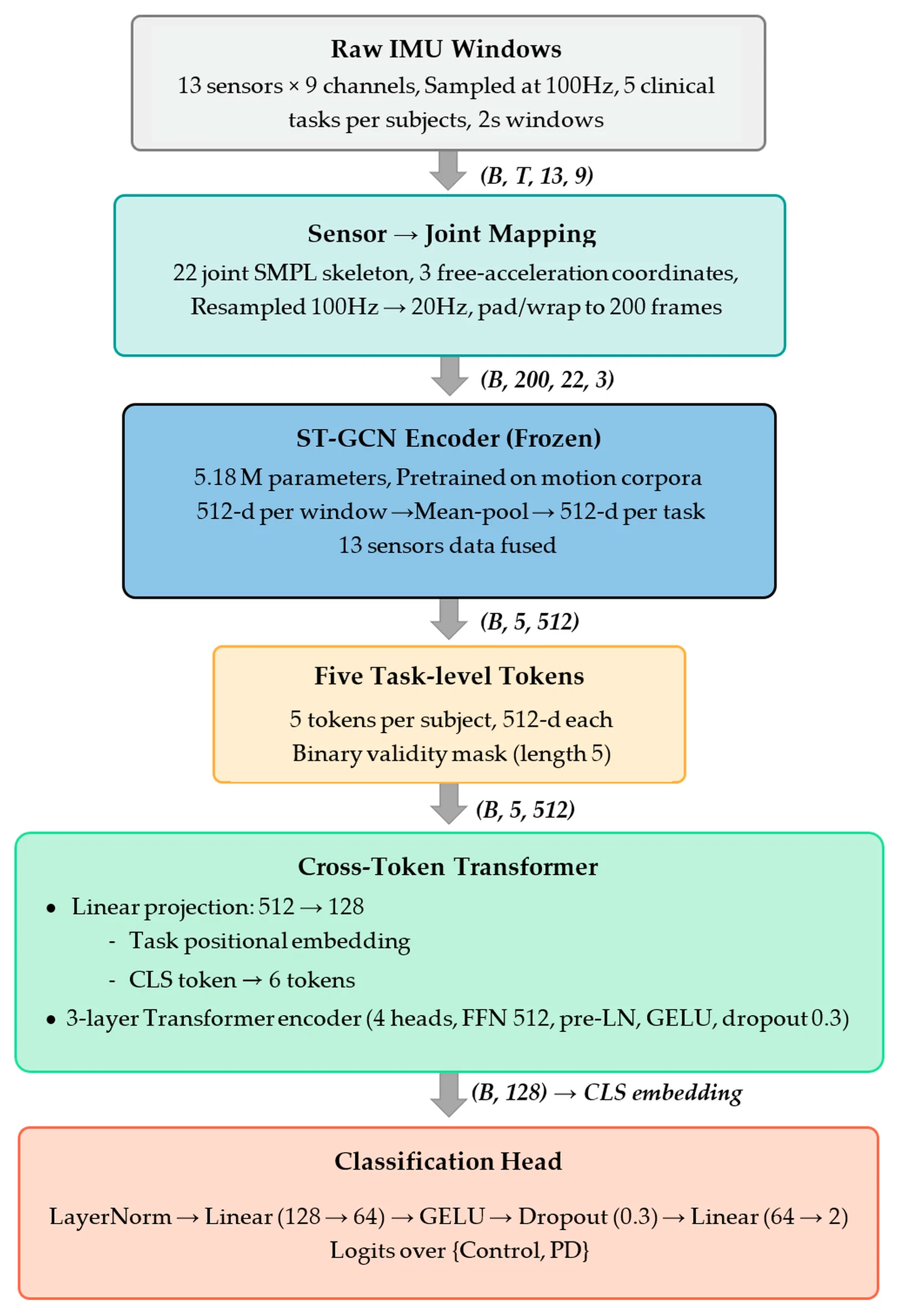
Overview of the proposed pipeline. Raw IMU windows from 13 body-worn sensors across five clinical tasks are processed by mapping each sensor to its closest skeletal joint and passing the windows through the frozen pretrained ST-GCN encoder (Figure 3) to yield a task-level motion embedding. The five resulting task-level embeddings, together with a binary validity mask flagging which tasks the subject performed, are aggregated by a three-layer Transformer with a learnable CLS token into a subject-level embedding for binary classification.

The CLS token aggregates information from the five task tokens and is fed to a small MLP head (LayerNorm → Linear 128 → 64 → GELU → Dropout → Linear 64 → 2) producing logits over {control, PD}. The model contains approximately 0.68 million trainable parameters above the frozen 5.18-million-parameter encoder. The complete end-to-end pipeline, from raw IMU windows through the five task-level tokens to the final classification head, is summarized in Figure 4. We describe the model as parameter-efficient only in the sense that few parameters are updated during training; the effective model that must be stored and run at inference comprises the full 5.86 million parameters of encoder plus aggregator, and the train–test gap reported shows that the trainable head is nonetheless large enough to overfit a cohort of this size.

### 3.6. Component Ablations

Component ablations are evaluated under identical training and evaluation conditions, and under the repeated leakage-free protocol of Section 3.9. Three ablations each disable exactly one element of the proposed model so that the contribution of that element can be isolated, and a fourth control addresses the concern that a frozen random encoder is not by itself an adequate control for pretraining. To control for parameter count, the three single-element variants retain the same architectural skeleton as the proposed model: the ablated element is either re-initialized (the encoder weights), multiplied by zero (the positional embeddings), or bypassed at the forward pass (the cross-token attention), without removing the corresponding parameters from the optimizer.

1. Ablation A (ST-GCN encoder without pretrained weights): the ST-GCN encoder is re-initialized with random weights and kept frozen, instead of loading the pretrained motion-capture weights; the architecture, the sparse-skeleton input scheme, and the entire downstream pipeline are otherwise unchanged. So that this is a fair architectural match, every parameter is reset to its standard initialization, and the batch-normalization parameters are left at their default (gamma = 1, beta = 0) rather than perturbed, and the learnable edge-importance weights are reset to their architectural default of ones; the random encoder therefore differs from the pretrained encoder only in its weights. This variant isolates the contribution of pretraining, distinct from that of the ST-GCN architecture itself. Because a frozen random representation is non-adaptive and might, on its own, overstate the pretraining effect, we add a fourth control, Ablation A (trainable): a randomly initialized encoder of the same architecture trained end-to-end on the cohort. Comparing the pretrained encoder against both a frozen and a trainable random encoder isolates the pretraining effect from both the architecture and the freezing.
2. Ablation B (no positional embeddings): the task position embeddings are multiplied by zero. This isolates the contribution of explicit positional information beyond what is already encoded by the token construction and the validity mask.
3. Ablation C (no cross-token attention): the three-layer Transformer is bypassed, and the CLS embedding is replaced by a uniform mean pool over valid tokens. This isolates the contribution of learned attention-based fusion, with the caveat that replacing attention with a parameter-free mean also removes aggregation capacity; the two effects cannot be separated in this single ablation, so the measured effect is an upper bound on the contribution of attention specifically.

### 3.7. Comparison Models: Deep and Classical Baselines

To establish whether the deep architecture is genuinely required and how the proposed pipeline compares with alternative deep models, we evaluate two families of comparison models using the same subject-disjoint 5-fold splits. The first family is six classical machine learning baselines that consume the same standardized 512-dimensional task-level tokens as the proposed model, flattened to a 2560-dimensional subject vector (5 task tokens × 512 dimensions): L2-regularized logistic regression, linear- and RBF-kernel support vector machines, a random forest, a gradient-boosting classifier, and an XGBoost classifier. All use class-balanced weighting; their hyperparameters are given in Table 5. Each is implemented as a scikit-learn pipeline, so that every transform (standardization, classifier) is fitted strictly on the training subjects of the fold, ensuring no leakage into the held-out fold.

### 3.8. Training Procedure

All deep variants are trained with the AdamW optimizer [46] at learning rate 3 × 10^−4^ and weight decay 1 × 10^−3^, with gradient clipping at ∥g∥ ≤ 1, batch size 16, and a cosine learning-rate schedule [47] over a maximum of 200 epochs, with early stopping on the inner-validation balanced accuracy (Section 3.9), with patience 40. The classification loss is class-weighted cross-entropy to compensate for the 100 PD/81 control imbalance. All classical baselines are trained with fixed hyperparameters described in Section 3.7.

### 3.9. Evaluation Protocol

All experiments use stratified 5-fold cross-validation at the subject level, repeated over five independent random seeds, yielding 25 held-out estimates per model (15 for the two end-to-end models, which are more costly and are run over three seeds). For each fold, training tokens are standardized with a StandardScaler fitted only on training subjects; the scaler is applied to the held-out fold, and the validity mask is reapplied so that absent tokens remain exactly zero. Model selection is nested and leakage-free: within each training fold, a stratified inner validation partition (15% of the training subjects, disjoint from the test fold) is held out, early stopping is performed on the inner-validation balanced accuracy, and the test fold is used only once, for final reporting. This removes the coupling between model selection and the reported estimate. We report four metrics: balanced accuracy (primary, robust to the mild class imbalance) [48], accuracy, macro-F1, and ROC-AUC, all computed at the subject level on each held-out fold. Balanced accuracy is the mean of the per-class recalls; accuracy is the overall fraction of correctly classified subjects; macro-F1 is the unweighted mean of the per-class F1 scores; and ROC-AUC is the area under the receiver operating characteristic curve computed from the predicted PD probabilities. Means and standard deviations are computed across all seed × fold estimates. Because repeated cross-validation produces correlated estimates (folds share training data), we assess differences between the proposed model and every other model with the Nadeau–Bengio corrected resampled paired *t*-test, which inflates the variance by a factor accounting for the train/test overlap and is our headline test; we additionally report an across-seed paired *t*-test (each seed contributing one fold-averaged estimate) and, for reference only, the naive paired *t*-test and Wilcoxon signed-rank test, which are anti-conservative on correlated folds and should not be read as the primary inference. Statistical significance is reported at the 0.05 level on the corrected test. The classical baselines are unaffected by the model-selection change, as they use fixed hyperparameters and no early stopping.

## 4. Results

### 4.1. Performance of the Full Model and Component Ablations

Under the leakage-free nested protocol with repeated cross-validation (five seeds, 25 held-out estimates), the proposed model attains a balanced accuracy of 0.834 ± 0.087, an accuracy of 0.825 ± 0.089, a macro-F1 of 0.842 ± 0.094, and an AUC of 0.842 ± 0.103, and is the top-performing model on balanced accuracy, accuracy, and macro-F1 (Table 6). Relative to the single-split protocol (0.847 ± 0.037), this leakage-free estimate is marginally lower and more variable, consistent with the removal of model-selection coupling and with honest fold-to-fold variability.

The drops are therefore asymmetric: the pretrained weights account for most of the deep model’s performance relative to its ablated variants, while the other two components contribute smaller gains. Among the six classical baselines, the three tree-based learners are the strongest: XGBoost (balanced accuracy 0.814 ± 0.069) and random forest (0.813 ± 0.083) trail the proposed model by 2.0 and 2.1 percentage points, and gradient boosting (0.807 ± 0.096) by 2.7 points; none of these gaps is statistically significant under the corrected test. On AUC, three comparison models exceed the proposed model’s 0.842 ± 0.103: random forest achieves the single highest AUC of any model (0.898 ± 0.070), followed by gradient boosting (0.861 ± 0.096) and the Spectrogram-CNN (0.838 ± 0.083); random forest’s AUC advantage does not reach significance under either the conservative corrected test (*p* = 0.47) or the across-seed test (*p* = 0.11). The single deep baseline trained from raw windows reaches balanced accuracy 0.778 ± 0.055, and the remaining classical baselines trail furthest (RBF SVM 0.760 ± 0.062, logistic regression 0.751 ± 0.056, and linear SVM 0.721 ± 0.083, lagging the proposed model by 7.4, 8.3, and 11.3 percentage points, respectively).

Each component ablation reduces balanced accuracy (Table 7): removing the pretrained weights (the frozen randomly initialized encoder of Ablation A) yields 0.679 ± 0.083 (a 15.5 percentage-point drop), and a trainable randomly initialized encoder yields 0.630 ± 0.088 (a 20.4-point drop, lower still because a from-scratch encoder overfits this cohort); removing the positional embeddings (Ablation B) yields 0.809 ± 0.088 (2.5 pp), and replacing the cross-token attention with mean pooling (Ablation C) yields 0.809 ± 0.095 (2.5 pp). Under the corrected paired test, the two random-encoder pretraining ablations are the only differences from the proposed model that reach significance on balanced accuracy (frozen *p* = 0.043, trainable *p* = 0.014); the trainable-random control shows a larger point difference (+0.213) and also clears the correction (*p* = 0.014), while the positional-embedding and attention ablations move balanced accuracy by amounts within the model’s own 0.087 standard deviation and are not significant.

### 4.2. Comparison with Deep and Classical Baselines

The proposed deep model leads every comparison model on balanced accuracy, accuracy and macro-F1 on this cohort, though by a smaller margin than the deep–classical gaps reported in some earlier wearable PD literature, and is exceeded on AUC by three of them (random forest, gradient boosting, and the Spectrogram-CNN). After correcting for the correlation between cross-validation folds, none of these between-model differences is statistically significant (Table 8); the comparison should therefore be read as an empirical ordering, not as a demonstration of superiority. Three observations frame it.

First, the proposed model leads all seven comparison models on balanced accuracy, accuracy and macro-F1. The linear baselines trail furthest (linear SVM 0.721 ± 0.083, logistic regression 0.751 ± 0.056, and RBF SVM 0.760 ± 0.062, lagging by 11.3, 8.3 and 7.4 percentage points in balanced accuracy), indicating that a linear-in-features model captures less of the structure in the task-level motion embeddings than the cross-token Transformer. The Spectrogram-CNN trained from raw windows also trails 0.778 ± 0.055, so a purpose-built deep model learned from scratch on this cohort does not match the transferred representation on threshold metrics, despite consuming all nine inertial channels. The three tree-based baselines are the strongest comparison models but still trail (XGBoost 0.814 ± 0.069, random forest 0.813 ± 0.083, and gradient boosting 0.807 ± 0.096, lagging the proposed model by 2.0, 2.1, and 2.7 percentage points in balanced accuracy); under the corrected test, these gaps are not significant (*p* = 0.76, 0.80, and 0.75). On AUC, however, random forest reaches 0.898 ± 0.070, exceeding the proposed model’s 0.842 ± 0.103 by 5.6 percentage points; gradient boosting (0.861 ± 0.096) and the Spectrogram-CNN (0.838 ± 0.083) also edge ahead of the proposed model on AUC. This AUC advantage of random forest does not survive either the conservative corrected test (*p* = 0.47) or the across-seed test (*p* = 0.11), so it is best read as a numerical ordering rather than a demonstrated difference.

Second, the strongest classical baselines (XGBoost, random forest and gradient boosting) reach a balanced accuracy of roughly 0.81 on the same standardized 512 d task-level tokens, confirming that the input representation by itself already carries substantial discriminative information for tree-based learners on tabular-style inputs in the low-to-moderate-N regime [30,47]. The 2.0- to 2.7-percentage-point gap to the proposed model indicates that the cross-token Transformer extracts at most modest additional discriminative structure beyond what tree-based learners recover from the token vector, and under the corrected paired test, this gap is not statistically significant; because the classical baselines now receive the same 2560-dimensional token vector as the proposed model (Section 3.7), the gap is no longer inflated by the earlier extreme input width, and the honest reading on this cohort is that the deep and strongest classical models are statistically indistinguishable. Random forest’s higher AUC suggests that its ranking of subjects by predicted PD probability is sharper, even though its threshold-dependent metrics are lower; because AUC is the threshold-independent measure, this divergence cautions against reading the proposed model’s lead on balanced accuracy as a uniformly stronger classifier.

Third, the spread within the classical family (XGBoost 0.814 vs. linear SVM 0.721, a 9.3 percentage-point range in balanced accuracy) is wider than the gap between the proposed model and the strongest classical baseline (2.0 percentage points, not statistically significant). Choice of classical learner therefore matters more than the deep-vs.-classical axis on this cohort, a result that depends on careful reporting against strong (not only linear) classical baselines.

### 4.3. Seed- and Fold-to-Fold Variability

Across the repeated protocol, the proposed model has a standard deviation of 0.087 on balanced accuracy over the 25 seed × fold estimates, appreciably wider than the 0.037 obtained from the single split; this increase is expected once the model-selection coupling is removed and variability is estimated across seeds as well as folds, and it is the reason the between-model differences of Section 4.1 and Section 4.2 are largely non-significant. The standard deviations of the other models are comparable (frozen-random Ablation A 0.083, positional-embedding Ablation B 0.088, attention Ablation C 0.095; classical baselines from 0.056 for logistic regression to 0.096 for gradient boosting). The per-estimate trajectories of the deep variants are correlated across folds, indicating that a non-trivial share of the variance reflects intrinsic subject-level difficulty rather than architecture-specific behavior, which is precisely why the corrected paired test (Section 3.9) that accounts for this correlation is the appropriate basis for inference.

### 4.4. Statistical Significance, Age, and Gender Analyses

Because the repeated protocol yields correlated estimates, we assess every difference from the proposed model with the Nadeau–Bengio corrected resampled paired *t*-test (headline) and an across-seed paired *t*-test (Section 3.9). Table 8 summarizes the decision-relevant comparisons. On balanced accuracy the differences that survive the corrected test are the proposed model versus the two randomly initialized encoders: the frozen control (mean difference +0.155, *p* = 0.043) and the trainable control (mean difference +0.213, *p* = 0.014). Both random-encoder controls are therefore separable from the proposed model even under the conservative correction. No comparison against a classical baseline, and neither of the positional-embedding or attention ablations, is significant on balanced accuracy. On AUC, random forest ranks subjects more sharply than the proposed model (difference −0.056); this advantage is not significant under the conservative corrected test (*p* = 0.47) or the across-seed test (*p* = 0.11). On AUC, the same pattern holds for the pretraining controls: the trainable random encoder is separated from the proposed model under the corrected test (+0.210, *p* = 0.015), while the frozen random encoder is not (+0.144, *p* = 0.149). The overall picture is therefore that the transfer (pretraining) effect is the one robust difference, and every remaining ordering, including random forest’s higher AUC, is within noise once fold correlation is accounted for.

Age. Because the control group is, on average, about 7 years older than the PD group, we tested directly whether age drives the discrimination. Using 1:1 nearest-neighbor caliper matching (±3 years), we obtained a matched subset of 114 subjects (57 PD/57 controls) with group mean ages that are essentially identical (PD 70.8 vs. control 71.1 years; balance test *p* = 0.84). On this matched subset, the balanced accuracy is 0.846, marginally higher than the full-cohort 0.838 under the same subject-level aggregation, rather than collapsing toward chance as an age-driven classifier would; a ±5 year caliper (*n* = 120) gives 0.842 (Table 9). Discrimination is also preserved within age bands, including the 65–75-year overlap band, where age cannot separate the groups (Table 9). Finally, within each class, the predicted PD probability is uncorrelated with age (controls Spearman rho = −0.03, *p* = 0.79; PD rho = 0.05, *p* = 0.60). Age imbalance is therefore acknowledged, but the evidence indicates that the discrimination is not explained by age.

The PD and control groups differ moderately in gender composition (PD 65/35 vs. control 35/46 M/F), so we considered whether gender rather than disease could drive the discrimination. Balanced accuracy, our primary metric, is the mean of the two per-class recalls and so is not inflated by class or subgroup imbalance by construction; combined with the class-weighted training loss, this limits the extent to which the gender imbalance alone could account for the reported performance. A quantitative per-gender performance breakdown, reported with proper across-seed dispersion, requires the prospectively balanced, multi-site replication already identified as necessary for the age confound, and is deferred to that setting.

## 5. Discussion

The combined evidence from Section 4.1, Section 4.2, Section 4.3 and Section 4.4 supports a specific and limited reading of the proposed pipeline on this cohort. With subject-disjoint folds and identical task-level tokens supplied to every model, and under the leakage-free repeated protocol with paired significance testing, the proposed deep model (i) is the top performer on balanced accuracy, accuracy and macro-F1, leading the strongest classical baseline (XGBoost) by 2.0 percentage points in balanced accuracy, a gap that is not statistically significant; (ii) exceeds the component ablations by 2.5 to 20.4 percentage points in balanced accuracy, with the gap to the encoder-without-pretraining controls (15.5 pp frozen, 20.4 pp trainable) far larger than the gaps to the positional-embedding and attention ablations (2.5, 2.5 pp), and both pretraining gaps surviving the corrected test (frozen *p* = 0.043, trainable *p* = 0.014); and (iii) is exceeded on AUC by random forest (0.898 ± 0.070 vs. 0.842 ± 0.103), gradient boosting (0.861 ± 0.096) and the Spectrogram-CNN (0.838 ± 0.083), with random forest’s AUC advantage not significant under the corrected test (*p* = 0.47) or the across-seed test (*p* = 0.11). We read this evidence conservatively. The effects that are large relative to fold-to-fold noise, and the only ones significant under the corrected test, are the two pretraining ablations, so the claim we are confident in is a transfer-learning claim: a frozen motion-pretrained skeletal encoder carries most of the discriminative signal for multi-task wearable PD detection, and the majority of that signal comes from the pretrained weights rather than from the ST-GCN architecture alone. The remaining design elements, the positional embeddings and the cross-token attention, each move balanced accuracy by an amount within the full model’s 0.087 standard deviation, so on this cohort, their individual value cannot be separated from noise; they are retained because they are well-motivated and harmless, not because the present experiments demonstrate that each is necessary. The AUC result deserves equal emphasis rather than relegation. AUC is the threshold-independent summary of ranking quality, and random forest attains the numerically highest AUC (0.898 vs. 0.842); although this difference is not statistically significant, the proposed model cannot be claimed to be a uniformly stronger discriminator, since it leads to threshold metrics while random forest ranks subjects by predicted PD probability at least as sharply. Which model is preferable, therefore, depends on the intended use, a fixed-threshold screening decision versus a ranked triage list, and the discrepancy suggests the proposed model’s lead on threshold metrics may partly reflect where the default operating point falls on this particular split, a possibility that a calibration analysis and threshold sweep would be needed to confirm. Calibration analysis and a threshold sweep, listed in Section 5.4, are needed to settle this.

### 5.1. Component Contributions

The component ablations clarify what each part of the pipeline contributes. Removing the pretrained weights from the encoder is by far the most damaging: a frozen randomly initialized ST-GCN reduces balanced accuracy from 0.834 to 0.679 (a 15.5 percentage-point drop) and AUC from 0.842 to 0.699, and a randomly initialized encoder trained end-to-end reduces balanced accuracy further to 0.630 (a 20.4-point drop), because a from-scratch encoder overfits a cohort of this size. That the trainable random control is worse than the frozen one confirms the effect is not an artifact of freezing a random representation, and both random-encoder comparisons survive the corrected significance test (frozen *p* = 0.043, trainable *p* = 0.014). This is the dominant finding of the ablation study: the pretrained skeletal-motion weights, not the ST-GCN architecture in isolation, supply the majority of the discriminative information used by the model. The remaining two ablations cause much smaller drops: removing the positional embeddings drops balanced accuracy to 0.809 (2.5 percentage points), and replacing the cross-token attention with uniform mean pooling drops it to 0.809 (2.5 percentage points). Both drops are within the fold-to-fold standard deviation of the full model (0.087), and neither is statistically significant, so the strongest statement supported by this cohort is that the pretrained encoder dominates, while the contributions of the positional embeddings and the cross-token attention are individually unresolved. One implication should be stated plainly rather than glossed. Because the ST-GCN fuses the thirteen sensors and produces a whole-body embedding per task, a grid indexed jointly by task and sensor could only be built by replicating that embedding across sensors, and would carry no sensor-specific signal content; such a sensor axis would encode only which body locations contributed to a recording, through the validity mask and positional embeddings. We therefore do not use a sensor axis in the evaluated model: the proposed model aggregates the five task-level tokens directly (Section 3.3). A genuinely sensor-specific representation would require per-sensor encoding rather than a replicated whole-body embedding, and is identified as future work in Section 5.4. This also determines what the present architecture can and cannot say about sensor reduction, a question of direct practical importance for simplified hardware. Because the graph convolution fuses all thirteen sensors inside the frozen encoder, before any component the aggregator can inspect, the model exposes no per-sensor quantity: the cross-token attention map is defined over clinical tasks, not sensors, and therefore cannot be read as a sensor-importance ranking. A sensor-reduction result would require either retraining the encoder path with sensor subsets ablated at the input, or the per-sensor encoding scheme above. We consider it more useful to state this constraint explicitly than to offer an attention-derived sensor ranking that the architecture does not support.

### 5.2. Computational Complexity

It is useful to compare the computational cost of the proposed pipeline with that of the classical benchmarks, since cost is a practical consideration for any future clinical deployment. The proposed pipeline has two stages with very different cost profiles. The first is a one-off, inference-only forward pass of the frozen ST-GCN encoder over every valid IMU window; this stage carries the dominant parameter count (5.18 M) and the dominant arithmetic cost, which scales linearly with the number of windows and, per window, with the 200-frame length, the 22 graph joints, and the 64→512 channel schedule of the ten graph-convolutional blocks. Because the encoder is frozen, this stage is executed once per subject and never back-propagated through, so it contributes to training cost only as a fixed feature-extraction pre-pass. The second stage is the trainable head: a 512→128 projection, three pre-norm Transformer blocks, and a two-layer classifier, totaling only ≈0.68 M trainable parameters. The Transformer’s self-attention is quadratic in sequence length, but the sequence here is fixed at six tokens (five task tokens plus CLS), so the attention cost is a small constant (of order 6^2^ per layer per head) and is negligible next to the encoder pre-pass. Training therefore optimizes a sub-million-parameter head over precomputed embeddings, which is fast and modest in memory usage. The effective model that must be stored and executed at inference is nonetheless the full 5.86 M parameters (5.18 M frozen encoder plus 0.68 M head); the term “parameter-efficient” in this paper refers specifically to the number of parameters updated during training, not to the inference footprint, and should be read in that restricted sense. By comparison, the classical baselines operate directly on the 2560-dimensional flattened token vector and require no encoder pre-pass once the tokens exist, but the token vector itself is a product of the same ST-GCN feature extraction; the baselines are thus cheaper only in their classifier stage. Within that stage, logistic regression and the linear SVM are inexpensive (cost roughly linear in the 2560 features per subject); the random forest of 500 trees and the gradient-boosting ensemble of 200 trees are heavier at training time, scaling with the number of trees, the tree depth, and the feature count, though all remain trainable in seconds to minutes on a cohort of 181 subjects. In summary, the proposed model is not computationally expensive in its trainable part, and its principal cost, the frozen encoder pre-pass, is shared by the classical baselines that consume the same tokens; the architecture is therefore practical for the cohort sizes typical of clinical PD studies, and a precise wall-clock and FLOP comparison is included among the planned experiments in Section 5.4.

### 5.3. Significance and Practical Implementation

Taken at the level its evidence supports, the study advances the field in two concrete ways. First, it provides an ablation-grounded measurement, rather than an assumption, that a motion-capture-pretrained ST-GCN encoder transfers usefully to inertial PD assessment: the 15.5-to-20.4 percentage-point cost of removing the pretrained weights (frozen and trainable random controls, respectively), significant under the corrected test, is, to our knowledge, the first direct quantification of this transfer effect on a full body-worn IMU PD cohort, and it gives subsequent researchers a reason to start from pretrained motion encoders rather than training small clinical models from scratch. Second, the task-token representation offers a reusable way to organize heterogeneous multi-task wearable recordings into a single subject-level representation that downstream models, deep or classical, can consume; the validity-mask mechanism further allows the representation to tolerate missing tasks, which are common in real clinical data collection. We do not claim the sensor axis as a validated contribution, since, in this cohort, it carried no sensor-specific signal (Section 5.1); the reusable core is the transfer result together with the missing-data-tolerant task-token organization. For practical implementation, the parameter-efficient trainable head, the frozen and therefore reusable encoder, and the modest compute requirements (Section 5.2) make the pipeline realistic to train and run within the cohort sizes and hardware typically available to clinical research groups, and the structured representation is compatible with the kind of multi-task wearable protocols already used in movement disorder clinics. We are deliberately measured about the strength of these claims: the present results are obtained on a single-site, single-visit cohort of 181 subjects with the methodological caveats detailed below, so the appropriate statement is that the approach has clear potential for practical clinical decision support and a reusable methodological core, but that this potential is demonstrated at the pilot scale and requires the external, multi-site, multi-visit validation specified in Section 5.4 before any deployment claim could responsibly be made.

### 5.4. Limitations and Future Work

We state the principal limitations and pair each with the specific experiment each motivates. Some are addressed by the protocol adopted here; the remainder bound the interpretation and are stated as such rather than deferred wholesale to future work.

1. Single-seed evaluation and model-selection coupling (addressed). The single-seed evaluation reported one seed, one 5-fold partition, and an early stopping protocol that selected the stopping epoch on the test fold. The protocols adopted here avoids both: it is now runs over five independent stratified splits (25 per-model estimates), model selection uses a nested inner-validation partition disjoint from the test fold, and differences are assessed with the Nadeau–Bengio corrected paired test and an across-seed test (Section 3.9 and Section 4.4). Removing the coupling lowered the headline balanced accuracy from 0.847 to 0.834 and widened its standard deviation, as anticipated, and the corrected tests show that only the pretraining effect is separable from noise.
2. Single-site, single-visit cohort (remains). WearGait-PD is a single-site, single-visit cohort of 181 subjects, modest for deep learning, and the proximate cause of both the train–test gap and the wide variances. A single visit precludes longitudinal validation, a single site leaves device- and population-specific confounds unestimated, and the binary label without severity stratification or differential-diagnosis controls means the results should not be read as evidence of specificity against confounding movement disorders. Two design choices also remain to be optimized and are future work: the model uses only the three free-acceleration channels of the nine available per sensor, and it maps the thirteen sensors onto the encoder’s 22-joint skeleton with nine joints zero-filled; whether populating all channels, or a denser skeleton, transfers better cannot be settled here. A per-sensor encoding scheme, which would additionally enable a physical sensor-reduction study relevant to simplified hardware, is the natural vehicle for both.
3. Age imbalance (mitigated, external replication remains). The analyzed subset is not strictly age-matched: controls are, on average, about 7 years older than the PD group (Section 3.1). This is addressed directly in (Section 4.4) with 1:1 caliper matching, age-band subgroups, and within-class probability-age correlations, and find discrimination is preserved under matching (0.846 at a 0.3-year matched gap) and uncorrelated with age within each class, so the discrimination is not explained by age in this cohort. This does not remove the need for external validation. Future work: validation on an independent, multi-site cohort with multi-visit recordings, prospectively age-matched and differential-diagnosis controls, and severity labels is a precondition for any clinical interpretation and remains the most important non-computational follow-up.

This study delivers a reusable result: an ablation-grounded, significance-tested measurement of motion-pretrained encoder transfer to inertial PD detection, a missing-data-tolerant task-token representation, and an honestly benchmarked, leakage-free evaluation with a parameter-efficient trainable head. The evidence, however, is from a single-site, single-visit pilot cohort; the deep-classical margin is modest and not statistically significant; and a classical baseline attains the highest AUC, though not by a statistically significant margin. With nested and repeated cross-validation now in place, the remaining experiments required to convert this potential into a validated clinical contribution are external multi-site, multi-visit replication, a per-sensor encoding and sensor-reduction study, a full-channel encoder variant, and a calibration analysis with a threshold sweep.

## 6. Conclusions

We presented a pipeline for binary Parkinson’s disease detection from multi-task wearable IMU recordings whose central claim is representational rather than architectural: a frozen motion-capture-pretrained ST-GCN encoder produces task-level embeddings that are directly discriminative for PD, and these embeddings transfer effectively across multiple downstream classifier families. We demonstrate this in two ways: First, a parameter-efficient three-layer Transformer aggregator built on five task-level tokens of these embeddings achieves balanced accuracy: 0.834 ± 0.087, macro-F1 0.842 ± 0.094, and AUC 0.842 ± 0.103 on the WearGait-PD cohort (*n* = 181, 100 PD/81 controls) under a leakage-free nested protocol with repeated subject-disjoint stratified 5-fold cross-validation, with only ≈0.68 M trainable parameters above the frozen 5.18 M-parameter encoder. Second, six classical machine learning baselines fed the same ST-GCN-bearing tokens also reach strong performance: random forest 0.813 (AUC 0.898), XGBoost 0.814, and gradient boosting 0.807 in balanced accuracy, whereas removing the pretrained weights from the encoder collapses balanced accuracy by 15.5 percentage points when frozen and 20.4 when trained end-to-end, two differences that are significant under the corrected test. The convergence of these two observations identifies the pretrained ST-GCN embedding, rather than the surrounding aggregator, as the component that carries the signal. The remaining design elements, the positional embeddings and the cross-token attention, each move balanced accuracy by only 2.5 percentage points, within the model’s 0.087 fold-to-fold standard deviation and not statistically significant, and we explicitly do not claim them as validated contributions on this cohort; nor do we claim the sensor axis, which, in this cohort, carried no sensor-specific signal. At this cohort size, the deep aggregator and the random forest classifier are statistically indistinguishable on every metric under the corrected test, so we do not designate a single winner: the deep model leads on threshold metrics and the random forest attains the highest AUC, both within noise. With leakage-free nested model selection, repeated-seed cross-validation, and paired significance testing in this revision, we set out in Section 5.4 the remaining experiments, external multi-site, multi-visit replication, per-sensor encoding and sensor-reduction study, full-channel encoder variant, and calibration analysis with threshold sweep, which are required to convert these descriptive pilot findings into validated clinical claims.

## Figures and Tables

**Table 1:** Recently published binary Parkinson’s disease vs. control detection studies using wearable inertial or pressure sensors, with the present work for context.

Study	Year	Cohort	Sensors	Model	Reported Performance
Williamson et al. [41]	2021	UK Biobank (≈1900 PD + age-matched HC)	Single wrist accelerometer	Classical ML (gait + low-movement fusion)	AUC 0.85 (fused features)
Varghese et al. [42]	2024	PADS (276 PD, 79 HC, 114 DD)	Two wrist smartwatches (3-axis acc.)	Deep learning (CNN) + classical fusion	Balanced acc. 0.912 (PD vs. HC)
Adams et al. [43]	2024	WATCH-PD (82 early PD, 50 HC)	Smartwatch + smartphone (multi-modal)	Random forest	Acc. 0.923, Sens. 0.900, Spec. 1.000
Nanayakkara et al. [40]	2025	WearGait-PD subset (39 PD, 38 HC)	Pressure insoles (CoP features)	Logistic regression (23 features)	Acc. 0.875, ROC-AUC 0.921

**Table 2:** Demographic and clinical summary of the WearGait-PD cohort used in this study (*n* = 181). Continuous variables are given as mean ± standard deviation. PD duration and modified Hoehn and Yahr (H&Y) stage are reported for PD subjects only.

Characteristic	PD	Control
Subjects	100	81
Age (Years)	67.0 ± 8.3	74.7 ± 8.6
Age range (Years)	45–83	47–91
Gender, M/F	65/35	35/46
Weight (kg)	78.1 ± 17.3	80.9 ± 17.2
PD duration (Years)	7.5 ± 5.9	—
H&Y stage	2.2 ± 0.6	—

**Table 3:** Clinical-task coverage in the WearGait-PD cohort used in this study (*n* = 181). All five tasks are present in at least 98% of subjects. Rare absences are handled at the token level through a binary validity mask.

Clinical Task	Subjects with Task	Total Cohort	Coverage
Self-paced walking	181	181	100%
Timed up-and-go (TUG)	180	181	99%
Hurried-pace walking	181	181	100%
Tandem gait	178	181	98%
Quiet-standing balance	178	181	98%

**Table 4:** Mapping of the 13 body-worn IMU sensors to joints of the 22-joint skeletal graph used by the pretrained ST-GCN encoder. The 9 joints not listed (indices 0, 10, 12, 14, 15, 16, 18, 19, 20) carry no sensor and are zero-filled in every window.

Sensor	Joint Index	Anatomical Region
LowerBack	9	Lower trunk/lumbar spine
Xiphoid	11	Upper trunk/sternum
Forehead	13	Head
R_Wrist	21	Right wrist
L_Wrist	17	Left wrist
R_MidLatThigh	1	Right thigh
L_MidLatThigh	5	Left thigh
R_LatShank	2	Right shank
L_LatShank	6	Left shank
R_Ankle	3	Right ankle
L_Ankle	7	Left ankle
R_DorsalFoot	4	Right foot
L_DorsalFoot	8	Left foot

**Table 5:** Hyperparameters of the six classical baselines. All learners use class-balanced weighting; the two support vector machines additionally use Platt (sigmoid) probability calibration.

Model	Key Hyperparameters
Logistic regression (L2)	L2 penalty, regularization strength *C* = 0.1
SVM (linear kernel)	*C* = 0.1, probability calibration
SVM (RBF kernel)	*C* = 1.0, scale gamma, probability calibration
Random forest	500 trees, minimum split size 4
Gradient boosting	200 stage-wise trees, maximum depth 4, learning rate 0.05
XGBoost	300 trees, maximum depth 5, learning rate 0.05, 80% row and feature subsampling

**Table 6:** Subject-level 5-fold cross-validation results on the WearGait-PD cohort (*n* = 181), comparing the proposed model with end-to-end deep baselines Spectrogram-CNN and six classical baselines. Values are mean ± standard deviation across all seed × fold estimates under the repeated leakage-free protocol (5 seeds × 5 folds = 25 estimates per model; 15 for the Spectrogram-CNN, which is run over 3 seeds); the proposed model’s leading metrics are shown in bold. The proposed model is the top performer on balanced accuracy, accuracy, and macro-F1, but is exceeded on AUC by random forest, gradient boosting, and Spectrogram-CNN.

Model	Balanced Accuracy	Accuracy	Macro-F1	AUC
Proposed Model	**0.834 ± 0.087**	**0.825 ± 0.089**	**0.842 ± 0.094**	0.842 ± 0.103
Spectrogram-CNN	0.778 ± 0.055	0.776 ± 0.047	0.764 ± 0.056	0.838 ± 0.083
SVM (linear)	0.721 ± 0.083	0.714 ± 0.086	0.709 ± 0.089	0.752 ± 0.102
SVM (RBF)	0.760 ± 0.062	0.754 ± 0.061	0.752 ± 0.062	0.798 ± 0.068
Logistic Reg. (L2)	0.751 ± 0.056	0.746 ± 0.057	0.743 ± 0.057	0.835 ± 0.069
Random Forest	0.813 ± 0.083	0.809 ± 0.081	0.780 ± 0.085	0.898 ± 0.070
XGBoost	0.814 ± 0.069	0.810 ± 0.068	0.802 ± 0.072	0.820 ± 0.068
Gradient Boosting	0.807 ± 0.096	0.803 ± 0.096	0.796 ± 0.099	0.861 ± 0.096

**Table 7:** Subject-level cross-validation results of the component ablations on the WearGait-PD cohort (*n* = 181), under repeated stratified 5-fold cross-validation (5 seeds; 25 held-out estimates per token model, 15 for the trainable end-to-end control). Values are mean ± standard deviation across all seed × fold estimates. Ablation A re-initializes the ST-GCN encoder with random weights and keeps it frozen; Ablation A (trainable) trains a randomly initialized encoder of the same architecture end-to-end; Ablation B removes the task positional embeddings; and Ablation C replaces the cross-token attention with uniform mean pooling. The proposed model’s metrics are shown in bold for reference.

Model	Balanced Accuracy	Accuracy	Macro-F1	AUC
Proposed Model	**0.834 ± 0.087**	**0.825 ± 0.089**	**0.842 ± 0.094**	0.842 ± 0.103
Ablation A: ST-GCN encoder without pretrained weights	0.679 ± 0.083	0.682 ± 0.082	0.675 ± 0.085	0.699 ± 0.110
Ablation A (trainable): random encoder trained end-to-end	0.630 ± 0.088	0.637 ± 0.090	0.590 ± 0.111	0.655 ± 0.094
Ablation B: positional embeddings removed	0.809 ± 0.088	0.805 ± 0.091	0.802 ± 0.091	0.766 ± 0.083
Ablation C: attention replaced with mean pooling	0.809 ± 0.095	0.809 ± 0.095	0.806 ± 0.096	0.800 ± 0.074

**Table 8:** Significance of the difference between the proposed model and selected comparison models under repeated cross-validation. Delta is the mean of the proposed-minus-comparison score differences across all seed × fold pairs. *p* (corrected) is the Nadeau–Bengio corrected resampled paired *t*-test; *p* (across-seed) treats each seed as one fold-averaged estimate. An asterisk marks significance at the 0.05 level on the corrected test.

Proposed vs.	Metric	Δ	*p* (Corrected)	*p* (Across-Seed)
Ablation A (frozen random)	Balanced acc.	+0.155	0.043 *	<0.001
Ablation A (trainable random)	Balanced acc.	+0.213	0.014 *	0.030
Ablation B (no pos. emb.)	Balanced acc.	+0.025	0.731	0.366
Ablation C (mean pooling)	Balanced acc.	+0.025	0.771	0.294
Random forest	Balanced acc.	+0.021	0.779	0.402
Gradient boosting	Balanced acc.	+0.027	0.734	0.189
XGBoost	Balanced acc.	+0.020	0.788	0.532
Ablation A (frozen random)	AUC	+0.144	0.149	0.014
Ablation A (trainable random)	AUC	+0.210	0.015 *	0.019
Ablation B (no pos. emb.)	AUC	+0.076	0.402	0.029
Ablation C (mean pooling)	AUC	+0.042	0.448	0.069
Random forest	AUC	−0.056	0.473	0.113
Gradient boosting	AUC	−0.018	0.839	0.226
XGBoost	AUC	+0.022	0.755	0.337

**Table 9:** Balanced accuracy of the proposed model under age matching and within age bands, computed from subject-level mean predicted probabilities aggregated across seeds. Mean ages are shown where applicable.

Subgroup	Subjects (*n*)	Mean Age PD/Ctrl (yr)	Balanced acc.
Full cohort	181	67.0/74.7	0.834
Age-matched (±3 yr)	114	70.8/71.1	0.846
Age-matched (±5 yr)	120	70.7/71.4	0.842
Age < 65	45	—	0.804
Age 65–75	77	—	0.791
Age > 75	59	—	0.905

## Data Availability

This study analyzed the publicly available WearGait-PD dataset, accessible through the Synapse platform at https://www.synapse.org/Synapse:syn52540892/wiki/ (accessed on 20 February 2026).

## Abbreviations

The following abbreviations are used in this manuscript:

AUC	Area under the receiver operating characteristic curve
CLS	Classification (token)
CV	Cross-validation
GELU	Gaussian error linear unit
IMU	Inertial measurement unit
MDS-UPDRS	Movement Disorder Society–Unified Parkinson’s Disease Rating Scale
MHSA	Multi-head self-attention
MLP	Multi-layer perceptron
PD	Parkinson’s disease
ST-GCN	Spatio-temporal graph convolutional network
TUG	Timed up-and-go (test)
